# Her Heart's Mistake

**Published:** 1889-06-22

**Authors:** E. D. Cumming

**Affiliations:** Author of "An Ordeal by Silence"


### Her Heart's Mistake.

By E. D. Cumming, Author of "An Ordeal by Silence."

(*Continued from page 158.*)

### Chapter X.—Mr. Warren.

"The rains are all very well," remarked Captain Phelps to Mrs. Arkwright one evening, "but now we have had a month of them, and the thing is growing monotonous."

"The damp heat is infinitely worse than the dry," said the lady, waving her fan lazily. "I wish I could persuade Jack to let me go up to Mussoorie."

"It's every bit as wet up in the hills. Bairdsley is all anxiety to come down again, Mrs. Brent tells me."

"I can't imagine anyone—even a man in love—wanting to leave Simla."

"Bairdsley is peculiar, then. He wants to come back and marry at once."

"And the lady doesn't see it?"

"The lady is firm in her determination to wait until the cold weather."

"That's rather odd. Kate can't be aware of the temptations which beset a man like her intended at a place like Simla. I could quite understand her being anxious to land such a fish, but I don't see why he should be in such a hurry."

"I don't think Bairdsley's is a fickle nature," said Captain Phelps, coldly, "and Miss Harrison would be the last to try and hasten their marriage from mercenary motives. She has too much pride."

"I didn't mean Miss Harrison particularly," explained Mrs. Arkwright, a little awkwardly; "I meant in a general way. Girls come out to India to marry, and one grows to look upon matrimony from a business-like point of view perhaps."

"Perhaps," assented Captain Phelps, briefly.

It was certainly the case that Captain Bairdsley had displayed great eagerness to return to the plains and lead Miss Harrison to the altar; and Mrs. Brent had made no secret of it, thinking, with great justice, that it proved his appreciation of the prize he had won in her niece. But it was out of the question for him to leave the hills yet; he had had a return of his fever whilst there, and had been laid up every alternate day for nearly a week with mild attacks of it.

"I was anxious to see whether it would have returned or not," said Dr. Harrison when the news reached them; "the twenty-eighth day is the critical one. For some occult reason these attacks come exactly four weeks after each other, to the day."

The scientific phenomenon her father referred to had little interest for Kate. It was enough for her to know that her lover was ill again, and she was restless and miserable until he wrote to announce his recovery. Their correspondence had been steadily maintained with spontaneity and warmth on both sides. George had told her that he was a bad correspondent that evening when they had had the conversation about his father, but the regularity and frequency of his letters would have satisfied the most exacting of engaged young ladies. He told her where he had been, at whose houses he had lunched and dined; of the afternoon "squashes" he had attended, and what songs he had sung there. Kate knew by name all the people he had met, and all the places whither Simla society resorted for its picnics. He kept her minutely informed of all the amusements and entertainments in which he had taken part since he had recovered strength sufficiently to go out; and, thanks to a sound constitution with which he had never taken liberties, he had been able to hire ponies and move about, a fortnight after he took up his quarters in the Rockcliff Hotel.

Kate was able to tell herself from day to day what George was doing, or where he was going, and would draw mental pictures of the houses he described. She knew exactly what all his movements were, and assured herself that no literary talent could tell her more. But there was in her inmost heart a vague, undefined consciousness that something her soul yearned for was absent; a misty feeling that she was not in touch with him; that his letters did not come home to her as she had fancied they would do. She had no standard of comparison; no other man had ever stood for an hour in the niche George Bairdsley occupied, and she sought to explain away the nameless longing which possessed her by the eagerness she felt to have him with her again. It could be only that, she thought. His letters were as warm and tender as his words had ever been. He wrote her such letters as she could read aloud to her father and aunt—for Bairdsley was not given to mawkish sentiment and reiterated endearments in every line, nor would such have been food to her hungry mind. Then what was this absent strain she missed? She could not tell; but it existed, and she could not disguise from herself that the feeling was akin to one of disappointment.

She was too close an observer not to have discovered in the near intercourse to which her lover's long illness gave rise that he was a man of somewhat superficial character, possessing no remarkable depth of thought. His sympathies were narrow in their range, and he was unquestionably selfish, and inconsiderate to all except her. But she had taught herself to believe that she would not have him otherwise; that he was her ideal in the rough, and that it would be her delight and privilege to smooth down and soften what she knew to be his bad points. She had summed up and weighed him, knowing he was not perfection, and his very faults and weaknesses, petty though they were, were dear to her.

She missed him very sorely, and found her chief pleasure in writing to him. She had not the fund of gaieties, lunches, picnics, dinners, dances, and concerts to draw upon that he had; but her letters were longer than his in spite of it, for she had the talent, never suspected by herself before, of making an interesting epistle out of nothing. Her letters were characteristic, and Bairdsley felt when he read them that she was as near him as though he were at her side, in the verandah of the bungalow down at Meerut. How she found so much to say was a pleasant puzzle he often asked her to solve; telling himself, as he did so, that this girl loved him with all her soul.

Life at Meerut was moving on in its usual "rains" groove, with nothing but an occasional mild scandal or flirtation to enliven it. Captain Andrew's exclusive attentions to Mrs. Arkwright were making people shake their heads and wonder how the Major could permit it. Almost every married lady in the station had her special attaché, upon whose services she could rely for escort duty when she wanted a companion for her morning ride or drive. No one could find fault with that; it was usual and recognised. But really the Andrews-Arkwright combination was carried a little too far; and despite the excellent terms existent between the lady's husband and her "bow-wow," Meerut found much to condemn.

"I spoke to her about it very circumspectly only the other day," said Mrs. Tenterdale to a few friends at the club, "and she laughed in that childish way of hers, and told me that he was so useful with the children. Just fancy!"

"Qui s'excuse," began Mrs. Strachan, and stopped; either because her French was a trifle rusty, or because she made it a boast that she never discussed her neighbour's business.

"Yes, indeed!" chorused the little circle; "Mrs. Strachan is *quite* right."

And Mrs. Strachan blushed faintly, either with pleasure at her half-quotation being so promptly understood, or because she recollected that she never talked scandal.

"I should never *dream* of allowing Major Wilton to come about my bungalow in the free-and-easy fashion Captain Andrews does about hers," said Mrs. Markham of the Royal Artillery.

"Of course, you would never allow such a thing!" said everybody at once.

"Major Arkwright cannot be aware of the way she encourages him," said Mrs. Tenterdale.

"How can he be—always away hog-hunting or buck-shooting as he is?"

"He has no right to be surprised at it when he leaves her so much alone."

"Indeed, he has not. I don't blame her in the very *least*," said one warmhearted woman.

"There is every excuse for her," said Mrs. Strachan; who felt that in saying so she cancelled her first remark, and gave her conscience absolution.

And the jury, having entered its verdict of guilty, added this strong recommendation to mercy, and talked about something else.

"There goes Captain Phelps with Gerty Walford!" observed Mrs. Markham, as that gentleman cantered down the Mall at the side of a small dark-eyed girl on a country-bred pony. "This isn't the first time I have seen them together."

"Oh, Captain Phelps is always dangling after some girl or another," said Mrs. Tenterdale. "I know him of old."

"Men like that never do marry," said Mrs. Strachan. "I don't know on which side the fault lies, but it seems to be the case."

"The last girl but one very nearly caught him," said Mrs. Tenterdale. "Some people said that he actually proposed, and that she refused him, but I don't believe it."

"He is one of those men whom everyone likes, but who don't appear to have power to inspire deep feeling in any one woman," observed Mrs. Pringle. "They seem to have been created for the benefit of all."

"The man's a born flirt," said Mrs. Tenterdale, "and I sincerely hope he will be caught before long. He ought to make Captain Bairdsley his model."

"Yes, indeed he ought," agreed the chorus; and then, as Captain Bairdsley and his perfections had been very thoroughly talked over on many occasions ere now, an inclination manifested itself to discuss the contents of somebody's last "box out from home" instead of that gentleman; and he was dropped in favour of the absorbing topic of millinery.

It was band night, and while these things were engaging the interest of the ladies at the club, Mrs. Brent, Kate Harrison, and Mrs. Arkwright were sitting in the Doctor's carriage, which had drawn up in the gardens, and was as usual, surrounded by men.

"Who is the stranger, Mr. Wentworth?" enquired Mrs. Arkwright, who had a sharp eye for a new face. She indicated a short sturdily-built man of five or six-and-thirty, who was conversing with Mr. Pringle. His new solah topee, worn at this hour when Anglo-Indians discard that heavy inconvenient headgear for something more civilised, bespoke him a new arrival. He did not look like a soldier, was too old to be a newly-fledged civilian, and Mrs. Arkwright's lightly sleeping curiosity was fully aroused.

"He's a lawyer, named Warren," said Mr. Wentworth. "He has come out out to join Mr. Pringle's business here, and only arrived this morning."

"A new lawyer," said Mrs. Arkwright, approvingly. "I like barristers, they are so amusing. I suppose its something about their profession that makes them so."

"I believe that the atmosphere of Middle Temple and the Law Courts is singularly exhilarating," said Captain Andrews gravely: "you always notice its effect upon clients."

"He is not a good-looking man," said Mrs. Arkwright, ignoring her friend's remark.

The stranger had turned his face towards the party, and Miss Harrison glanced at him. He was not a handsome man by any means; but the square, clean-shaven chin, wide firm mouth, and heavy over-hanging brows, attracted attention and stamped the owner as a person of no ordinary character. Kate did not look very closely at him; there was only one form in the world she cared to see, and Mr. Warren's arrival and personality did not interest her in the least.

"Here is papa," she said, as the Doctor's figure in its white uniform appeared at the gate of the gardens. "I suppose he has only just got away from the hospital."

Dr. Harrison came towards the carriage, but as he passed Mr. Pringle that gentleman was observed to step forward and detain him, and Mr. Warren was almost at the same moment presented to him.

"Mr. Pringle seems to have been waiting to introduce that man to your father, Kate," said Mrs. Arkwright, whose watchful eye nothing escaped. "I wonder who this Mr. Warren is. Go and ask Mr. Pringle all about him for me, please, Captain Andrews."

Her attaché went upon his errand, and having exchanged with Mr. Pringle a few rather hackneyed remarks upon the weather, came back to enlighten his liege lady from the fertile resources of his imagination.

"He is a widower with twelve children, Mrs. Arkwright; all girls, ranging from twenty-five to nine years of age. They are to join him out here in the cold weather."

"What *is* the use of sending a man like that out here?" asked Mrs. Arkwright indignantly. "He won't be able to do anything."

The little lady looked upon all male additions to the station society from a practical standpoint, whereon she was was not entirely alone. A man was welcome in proportion to his accomplishments. If he were a rider, waltzer, and tennis player, he was admittedly "useful"; while the more sedentary individual who devoted himself to whist and billiards was not of much account. If he were of marriageable age, say over five-and-twenty, and had means, his possible value was enhanced in the eyes of matrons with daughters or young wives with sisters. Mrs. Arkwright expected her sister to join her in the course of the ensuing winter, and had taken no pains to conceal the fact that her interest in new arrivals owed its increased intensity to the prospective appearance of that relative in Meerut. Hence Captain Andrew's thoughtful but mendacious attempt to satisfy her as to Mr. Warren's eligibility.

"I told Pringle to ask him to call upon you soon," he added, "but I'm afraid he won't be able to just yet, as the Pringles are going up to Mussoorie, and Mr. Warren will look after the business while they are away."

"*I* don't want him to call," said Mrs. Arkwright. "What a young-looking man to have such a family."

Captain Andrews was still plying her with instructive but misleading details about Mr. Warren when she got out of the Harrisons' carriage to go over to the club; and a few minutes later the Doctor came up to join his sister and daughter.

"Do you recollect General Warren, Agnes?" he enquired of the former, "The man who had the command at Devonport nearly fifteen years ago."

Mrs. Brent remembered him, and her brother continued:

"That is his son whom you saw with Pringle. He has brought a letter of introduction from his father to me; so you may expect him to come and call before long."

"Why, is that the prig of a boy I knew, who was reading for the Bar ages ago?"

"The same. Pringle is an old friend of his father's, and young Warren—though he isn't so young now, by the way—has come out to try if he can't do better here than in England."

"Is he married?"

"No; he's a single man. Why did you think he was married?"

"Captain Andrews had some ridiculous story about his huge family to tell Mrs. Arkwright."

"If Mrs. Arkwright will insist upon making enquiries through that ingenious aide-de-camp of hers she will collect a marvellous fund of information."

"She has an eye upon every new-comer with a view to her sister, I suppose."

"Very likely; but she shouldn't be quite so candid on the subject," and the Doctor stepped into the carriage and told the coachman to drive home.

*(To be continued.)*

